# Anti-PD-1 antibodies, a novel treatment option for advanced chemoresistant pulmonary lymphoepithelioma carcinoma

**DOI:** 10.3389/fimmu.2022.1001414

**Published:** 2022-12-06

**Authors:** Na Zhou, Hui Tang, Shuangni Yu, Yi Lin, Yingyi Wang, Yuzhou Wang

**Affiliations:** ^1^ Department of Medical Oncology, Peking Union Medical College Hospital, Chinese Academy of Medical Sciences and Peking Union Medical College, Beijing, China; ^2^ Department of Pathology, Peking Union Medical College Hospital, Chinese Academy of Medical Sciences and Peking Union Medical College, Beijing, China; ^3^ Department of oncology, Centro Hospitalar Conde de Sao Januario, Estrada do Visconde de S. Januario, Macau, China

**Keywords:** lung cancer, Lymphoepithelioma-like carcinoma, rare subtype, efficacy, immunotherpay

## Abstract

**Background:**

Pulmonary lymphoepithelioma-like carcinoma (LELC) exhibits a unique immune microenvironment, including high PD-L1 expression and abundant infiltrating-immune cells. However, the availability of PD-1/PD-L1 inhibitors in patients with LELC is still not determined.

**Methods:**

A total of 36 cases of pulmonary LELC treated with PD-1/PD-L1 inhibitors were reviewed, including 10 cases from our institute and 26 cases included from the literature. The Kaplan-Meier method and log-rank test were utilized to analyze the survival outcomes of LELC patients receiving immunotherapy, and the factors related to immunotherapy response were further examined.

**Results:**

Of the 10 patients from our institute, the median age was 53.5 years, adrenal glands and distant lymph nodes were the most common metastatic sites, and 4 of 8 (50%) patients had a PD-L1 TPS ≥50%. The median progression-free survival and overall survival in patients from our institute and from the literature were 11.6 and 27.3 months, 17.2 months and not reached, respectively. In all 36 patients, the objective response rate was as high as 57.6%. Patients with higher PD-L1 expression were more likely to have a tumor response, but the association of PD-L1 expression with survival time remains to be determined.

**Conclusions:**

PD-1/PD-L1 inhibitors in patients with pulmonary LELC demonstrated a promising efficacy in retrospective cohorts, and deserve further validation in prospective studies administrating in front-line setting.

## Introduction

Primary pulmonary lymphoepithelioma-like carcinoma (LELC) is a rare subtype of non-small cell lung cancer (NSCLC) predominantly affecting younger non-smokers in Southeast Asia where nasopharyngeal cancer (NPC) prevails (1, 2). First reported in 1987, it is a non-keratinizing, poorly differentiated, pulmonary-originated squamous cell carcinoma associated with Epstein-Barr Virus (EBV) infection (3). Recently, a genetic study using whole-exome sequencing has revealed that pulmonary LELC possesses a distinct genomic profile from other lung cancers but shares similar alterations with NPC, including constitutive activation of inflammatory nuclear factor kappa B (NF-ĸB) signaling driven by EBV-encoded oncoprotein latent infection membrane protein 1 (LMP1) and crippled innate antiviral immunity due to losses of type I interferon (IFN) genes (4). In addition, programmed cell death-ligand 1 (PD-L1) upregulation in pulmonary LELC has been recognized as a major culprit to blame for undermining adaptive immune response (4, 5). Indeed, pulmonary LELC is uniquely featured by its inflamed environment but effective immune evasion.

Optimal treatment for advanced pulmonary LELC has not been well established. Our previous study had identified that platinum-based combination chemotherapy with or without radiotherapy could achieve good initial responses (6). However, the majority of advanced tumors eventually progressed from upfront treatments and there are limited therapeutic options to choose from after their resistance to chemotherapy. Actionable oncogenic driver mutations in NSCLC, such as epidermal growth factor receptor (EGFR) mutation and anaplastic lymphoma kinase (ALK) rearrangement, rarely exist in pulmonary LELC (7). PD-1 blockade or its combination with chemotherapy has been proven effective in treating patients with advanced NPC (8–10). Based on the histologic and genetic resemblance of pulmonary LELC to NPC (4, 5, 11), one might naturally surmise that PD-1/PD-L1 inhibitors would emerge as another promising weapon in the therapeutic arsenal against this rare tumor.

Yet, only a handful of case reports have explored the efficacy of anti-PD-1/PD-L1 antibodies in pulmonary LELC (12–21). The rarity of this lethal disease makes it impossible to conduct any convincing clinical trial for this matter. Here, we reported a cohort of ten patients who received anti-PD-1 therapy after routine management failed. To our knowledge, this represents the first and the largest cohort to test PD-1 inhibitors as late-line treatment in patients with advanced pulmonary LELC. We also performed a focused search of the literature with Pubmed to identify studies of blocking PD-1/PD-L1 in pulmonary LELC and attempted to determine predictors of efficacy from collected clinicopathological traits in this enriched population.

## Methods

### Patients

From July 2017 to September 2020, 10 patients received anti-PD-1 antibodies after progression from previous chemotherapy in the Centro Hospitalar Conde de Sao Januario (CHCSJ), Macau for advanced pulmonary LELCs. Data were collected from the hospital information system. All these cases were confirmed by Epstein-Barr encoding region (EBER) positivity. Otolaryngologists’ consultations with nasopharyngoscopy check-ups and imaging tests were applied to rule out NPC or other origins of LELCs. Flat dosing of nivolumab (240mg every 2 weeks) or pembrolizumab (200mg every 3 weeks) were given until progressive disease or intolerable toxicity. Tumor assessments were performed by computed tomography before the anti-PD-1 treatment and every 6 or 9 weeks thereafter. Treatment response was determined according to Response Evaluation Criteria in Solid Tumors version 1.1 (RECIST) (22). Progression-free survival (PFS) was calculated from the date of starting anti-PD-1 treatment to disease progression or death due to any cause. Overall survival (OS) was calculated from the date of starting anti-PD-1 treatment to death due to any cause.

### PD-L1 immunohistochemistry and scoring

PD-L1 expressions of 8 patients were assessed, based on sample deriving from primary tumor, by the PD-L1 immunohistochemical (IHC) staining 22C3 pharmDx assay (Dako North America, CarpinteriaCA) that has been approved as a companion diagnostic for use in non–small-cell lung cancer (23). The staining protocol used in this study was as described in the instructions for the commercial assay. Expression was scored using a tumor proportion score (TPS) which is defined as the number of positive tumor cells divided by the total number of viable tumor cells multiplied by 100%.

### Literature search

We conducted a literature search for reports of pulmonary LELCs in the Pubmed database and collected 191 studies. We then screened out 11 studies that focused on using PD-1/PD-L1 inhibitors monotherapy or their combination to treat advanced diseases. 10 studies were finally recruited after removing 1 case that was duplicated in the CHCSJ cohort. Studies that contained original information on clinical results of PD-1/PD-L1 inhibitor treatment were screened, including original researches and case reports. Clinicopathological factors, PD-L1 expression status, tumor response, and survival data were collected.

### Statistical analysis

The Mann-Whitney U test was used to analyze the relationship between PD-L1 expression and tumor responses to anti-PD-1/PD-L1 therapy. PFS and OS were assessed using Kaplan-Meier method. Patients were divided into two groups (low/high) according to their PD-L1 expression level and based on the optimal cut-off value of PFS calculated by the “survminer” package of R software. Univariate and multivariate analyses were applied to identify prognostic factors of PFS. R (version 3.6.1, http://www.r-project.org) was used for statistical analyses. Two-tailed value of p < 0.05 was considered to be statistically significant.

## Results

### Clinicopathological features of the CHCSJ cohort

**Figure 1:**
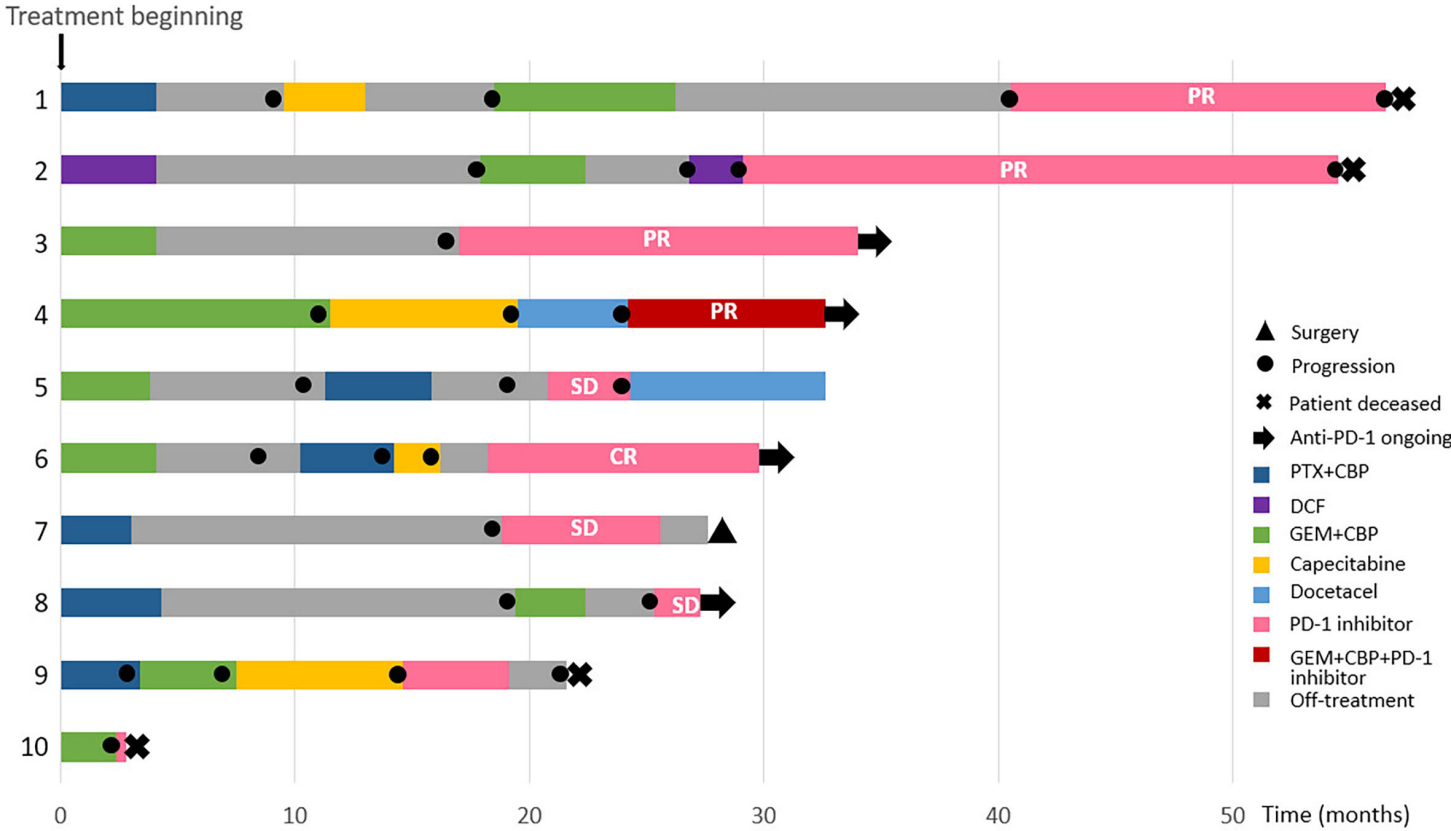
Summary of treatment reactions to chemotherapy and anti-PD-1 antibodies in the CHCSJ cohort.

The clinicopathological features of the CHCSJ cohort are described in **Table 1**. There were 4 males and 6 females. The median age of this cohort was 53.5 years (range, 46-67 years). Adrenal glands and distant lymph nodes were the most common sites of metastases (n=3, respectively). 8 patients had their PD-L1 expression tested: 4 (50%) were with TPS ≥50%, 3 with TPS 1~49%, and 1 with TPS <1%. 6 patients received pembrolizumab while 4 others nivolumab. Anti-PD-1 antibodies were applied in the 2^nd^ line setting in 3 patients, the 3^rd^ line setting in 3 patients, and the 4^th^ line setting in 4 patients. The median duration of anti-PD-1 treatment was 10.5 cycles (range, 1 to 30 cycles) and detailed descriptions of the duration of each treatment were shown in **Figure 1**.

**Table 1:** Clinicopathological factors of the CHCSJ cohort.

No.	Sex	Age	SmokingStatus	Stag-ing	TPS	Metastaticsites	Anti-PD-1 antibodies	No. of line	BestResponses	PFS(m)	OS(m)	Outcomes
1	F	63	Non-smoker	IVB	95%	Adrenals	Pembrolizumab	4^th^	PR	16.2	17.4	Deceased
2	F	50	Non-smoker	IVA	90%	Pleura	Pembrolizumab	4^th^	PR	25.4	27.3	Deceased
3	F	59	Non-smoker	IVB	35%	Lung & liver	Pembrolizumab	2^nd^	PR	17.5	17.5	Ongoing
4	F	67	Non-smoker	IVB	55%	Lung & NRLNs	Pembrolizuamb+GC	3^rd^	PR	8.5	8.5	Ongoing
5	M	59	Ex-smoker	IVB	NA	Bone & NRLNs	Nivolumab	3^rd^	SD	3.5	12.5	Progressed
6	M	60	Non-smoker	IVB	NA	NRLNs	Nivolumab	4^th^	CR	11.6	12	Ongoing
7	F	46	Non-smoker	IIIA	<1%	None	Pembrolizumab	2^nd^	SD	8.8	37	Surgery
8	F	48	Current-smoker	IIIB	10%	None	Pembrolizumab	2^nd^	SD	2	2	Ongoing
9	M	58	Current-smoker	IVB	20%	Pleura & liver	Nivolumab	4^th^	NA	3.5	3.5	Deceased
10	M	54	Current-smoker	IVB	95%	Adrenals & liver	Nivolumab	2^nd^	NA	0.3	0.3	Deceased

(NRLNs, Non-regional lymph nodes; GC, gemcitabine plus carboplatin; CR, complete response; PR, partial response; SD, stable disease; PD, progressive disease; NA, not applicable).

### Efficacy of PD-1 inhibitors in the CHCSJ cohort

8 patients were available for response rate analysis and 2 others died before an imaging-based assessment could be carried out. The objective response rate (ORR) was 62.5% (5/8), while disease control rate (DCR) was 100%. One male patient (12.5%) receiving nivolumab monotherapy as 3^rd^ line treatment achieved a complete response (CR). Partial response (PR) was reached in 4 (50.0%) patients who took pembrolizumab or its combination with chemotherapy in the 2^nd^ to 4^th^ line. 3 others achieved stable disease (SD).

During a median follow-up time of 18.5 months, 7 patients had progressed from PD-1 inhibitors or died from pulmonary LELC. The median PFS was 11.6 months (95% CI 8.8-NR [not reached]) and the median OS was 27.3 months (95% CI 17.4-NR) (**Supplementary Figure S1**).

### Efficacy of PD-1/PD-L1 inhibitors in the literature review

We finally recruited ten reports from 2017 to 2021 and gathered a total of 26 pulmonary LELC patients who were treated with PD-1/PD-L1 inhibitors for their unresectable or metastatic diseases (**Table 2**). 42.3% of patients received PD-1/PD-L1 inhibitors monotherapy, 34.6% of patients anti-PD-1 antibodies with chemotherapy, and 23.1% of patients anti-PD-1 antibodies with vascular endothelial growth factor receptor (VEGFR)-targeted tyrosine kinase inhibitors (TKIs). 19.2% of patients took immunotherapy in the 1^st^ line, 57.7% of patients in the 2^nd^ line, 23.1% of patients in the 3^rd^ or later lines. Of 25 patients whose responses had been clearly reported, 56% achieved PR and 40% SD. Only 1 patient experienced quick progression after nivolumab. The median PFS was 17.2 months (95% CI 7.7-NR). The median OS was not reached (**Supplementary Figure S2**).

**Table 2:** Summaries of the literature review.

No.	References	Sex	Age	SmokingStatus	Stag-ing	TPS	Anti-PD-1 antibodies	No. of line	BestResponses	PFS(m)	OS(m)	Outcomes
1	Xie, et al.	F	56	Non-smoker	IV	30%	Nivolumab+GEM	1^st^	SD	1	1	Lost f/u
2	Xie, et al.	F	49	Non-smoker	IIIB	60%	Nivolumab+GEM+ anlotinib	2^nd^	SD	6	6	Ongoing
3	Xie, et al.	M	48	Non-smoker	IVA	15%	Camrelizumab+ apatinib	3^rd^	SD	7	7	Ongoing
4	Qiu, et al.	F	56	Non-smoker	IVA	80%	Nivolumab	2^nd^	PR	4.4	4.4	Ongoing
5	Darrason, et al.	F	51	Ex-smoker	IVB	0%	Nivolumab	2^nd^	NA (PseudoPD)	7	14	Deceased
6	Kim, et al.	F	37	Non-smoker	IIIA	NA	Nivolumab	2^nd^	PD	0.8	1	Deceased
7	Kumar, et al.	M	56	Ex-smoker	IVA	NA	Nivolumab	4^th^	PR	21	25	Ongoing
8	Kumar, et al.	F	37	Non-smoker	IIIB	5%	Nivolumab	3^rd^	SD (PseudoPD)	24	27	Ongoing
9	Narayanan	F	76	Non-smoker	IVA	≥ 50%	Atezolizumab	2^nd^	PR	4	22	Deceased
10	Wu, et al.	F	58	Non-smoker	IIIA	40%	Sintilimab+anlotinib	2^nd^	PR	8.3	8.3	Ongoing
11	Wu, et al.	F	53	Non-smoker	IA2	30%	Pembrolizuamb+ nab-PTX	2^nd^	SD	10.9	10.9	Ongoing
12	Wu, et al.	F	48	Non-smoker	IV	90%	Pembrolizumab	2^nd^	SD	4.2	4.2	Lost f/u
13	Wu, et al.	F	56	Non-smoker	IVA	80%	Nivolumab	2^nd^	PR	7.5	15.3	Progressed
14	Wu, et al.	F	63	Non-smoker	IV	5%	Nivolumab+anlotinib	4^th^	SD	24.5	26	Ongoing
15	Tang, et al.	F	50	Non-smoker	IVB	10%	Nivolumab/Nivolumab+nab-PTX+NDP	2^nd^	SD (PseudoPD)	5	10	Progressed
16	Fu, et al.	M	68	Current-smoker	IVB	80%	Sintilimab	2^nd^	SD	3.4	3.4	Ongoing
17	Fu, et al.	F	56	Non-smoker	IVA	30%	Pembrolizumab	2^nd^	SD	7.7	7.7	Progressed
18	Fu, et al.	M	55	Non-smoker	IVB	90%	Pembrolizumab+ nab-PTX+CBP	1^st^	PR	9.5	11.8	Progressed
19	Fu, et al.	F	63	Non-smoker	IVB	70%	Pemrolizumab+ pemetrexel	1^st^	PR	14.4	14.4	Ongoing
20	Fu, et al.	F	70	Non-smoker	IVA	90%	Nivolumab+Anlotinib	4^th^	PR	15	15	Ongoing
21	Fu, et al.	M	46	Non-smoker	IVB	60%	Nivolumab+Apatinib	5^th^	PR	17	26.5	Progressed
22	Fu, et al.	F	56	Non-smoker	IVA	80%	Nivolumab+DXT	2^nd^	PR	17.2	17.2	Progressed
23	Fu, et al.	F	61	Non-smoker	IVB	1-50%	Sintilimab+Anlotinib	2^nd^	PR	6.4	6.4	Ongoing
24	Fu, et al.	F	54	Non-smoker	IVB	NA	Sintilimab+nab-PTX+CBP	1^st^	PR	7.5	7.5	Ongoing
25	Fu, et al.	M	43	Non-smoker	IIIC	80%	Sintilimab	1^st^	PR	3.2	3.2	Ongoing
26	Chen, et al	M	41	Non-smoker	IIIB	NA	Pembrolizumab+ nab-PTX+S1	2^nd^	PR	4.5	4.5	Progressed

(DXT, docetaxel; nab-PTX, Nanoparticle albumin-bound paclitaxel; GEM, gemcitabine; CBP, carboplatin; S-1 Triflurdine/tipiracil; NA, not applicable; PseudoPD, Pseudoprogression).

### Relation of PD-L1 expression with efficacy

**Figure 2:**
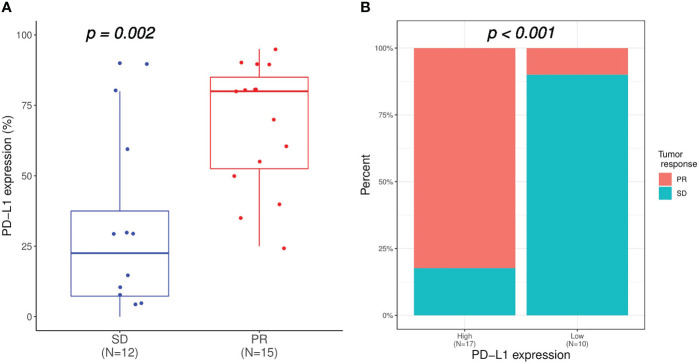
Relation of PD-L1 expression with immunotherapy response. **(A)** Comparison of PD-L1 expression between patients achieved SD and PR. **(B)** Comparison of immunotherapy response between patients with high (TPS ≥30%) and low (TPS <30%) PD-L1 expression. SD, stable disease; PR, partial response; TPS, tumor proportion score.

**Figure 3:**
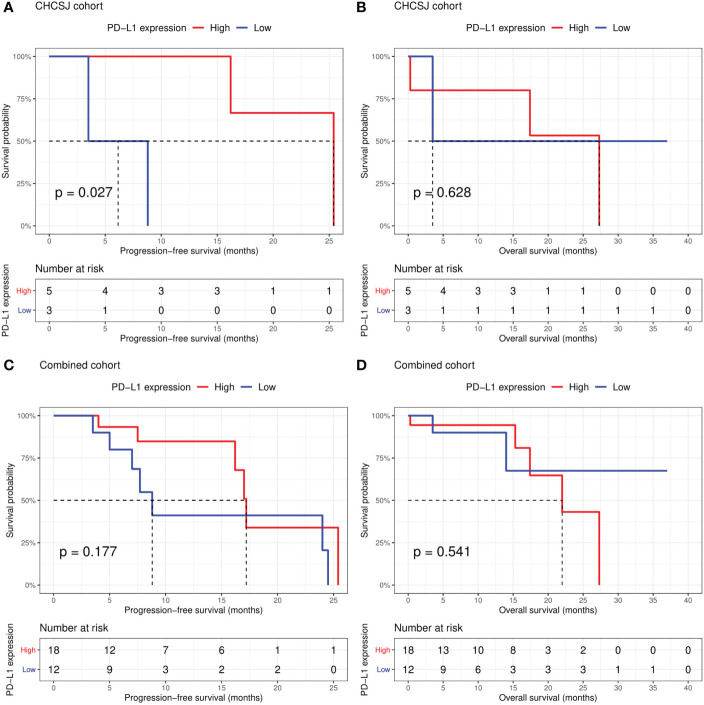
Comparison of progression-free survival **(A, C)** and overall survival **(B, D)** between patients with high (TPS ≥30%) and low (TPS <30%) PD-L1 expression in the CHCSJ and combined cohorts. TPS, tumor proportion score.

Altogether, the CHCSJ cohort and literature review contributed 36 pulmonary LELC patients. 30 patients had PD-L1 expression analyzed using TPS. 33 patients had clear tumor response data of anti-PD-1/PD-L1-based therapy. Of 27 patients with both available PD-L1 expression and immunotherapy response data, those who achieved PR had a significantly higher level of PD-L1 expression compared to those who achieved SD (median PD-L1 expression: 80% vs 22.5%, p=0.002, **Figure 2A**). Utilizing TPS 30% as the cut-off value determined by the “survminer” package of R software, patients with high PD-L1 expression (>30%) had higher ORR (14/17, 82.4% vs 1/10, 10.0%, p<0.001, **Figure 2B**) than those with low PD-L1 expression (≤30%). Likewise, the PFS of patients with high PD-L1 expression in the CHCSJ cohort was 25.4 months (95% CI 16.2-NR), significantly longer than that of those with low PD-L1 expression (6.2 months, 95% CI 3.5-NR, p=0.027, **Figure 3A**). However, such survival advantage in patients with high-level PD-L1 expression was not seen in OS analysis (27.3 vs 3.5 months, p=0.628, **Figure 3B**). Furthermore, there were no significant differences in PFS (17.2 vs 8.8 months, p=0.177, **Figure 3C**) and OS (22.0 months vs NR, p=0.541, **Figure 3D**) between those with high and low PD-L1 expression when the two cohorts combined.

### Prognostic relevance of clinicoprognostic factors for PFS and OS

During a median follow-up time of 10.5 months (range, 0.3-37 months), 18 patients experienced disease progression and 8 patients died of pulmonary LELCs among 36 patients. The Cox regression analysis was conducted to assess the impact of other clinical factors on PFS and OS. As shown in **Table 3**, univariate analysis showed that, compared to non-smoking patients, former or current smokers tended to have poor PFS (HR=12.08, p=0.053). However, neither smoking history nor treatment modality became independent predictors in multivariate analysis. In contrast, the difference in OS between never-smokers and ever-smokers was statistically significant (HR 30.74, 95% CI 2.11-447.25, p=0.012). However, other factors including sex, age (≥60y vs <60y), tumor stage (locally advanced vs metastatic), treatment modality (monotherapy or combination), liver metastasis (yes vs no), treatment scenario (<2^nd^ line vs ≥3^rd^ line) and PD-L1 expression (TPS >30% vs ≤30%) did not seem to exert any significant influence on the OS of patients with pulmonary LELCs (**Table 4**). Furthermore, compared with patients receiving immunotherapy alone, those who received immunotherapy combined with chemotherapy or targeted therapy had statistically non-significant improved PFS (8.8 vs 17.2 months, p=0.128) and OS (15.3 months vs NR, p=0.541, **Supplementary Figure S3**).

**Table 3:** Univariate and multivariate analyses of factors for progression-free survival in the combined two cohorts.

Variables	Univariate analysis		Multivariate analysis	
	HR (95% CI)	*p*	HR (95% CI)	*p*
Sex (Female vs. Male)	0.48 (0.17,1.35)	0.166	0.54 (0.18,1.59)	0.266
Age (≥60 vs. <60)	0.72 (0.23,2.24)	0.567	-	-
Smoking history (Yes vs. No)	12.08 (0.96,151.4)	0.053	6.26 (0.47,83.78)	0.166
TNM stage (IV vs. III)	0.67 (0.21,2.09)	0.487	-	-
PD-L1 expression (Low vs. High)	2.07 (0.64,6.68)	0.223	-	-
Multiple metastases (Yes vs. No)	0.9 (0.29,2.8)	0.852	-	-
Liver metastasis (Yes vs. No)	1.31 (0.41,4.14)	0.647	-	-
Treatment line (>2 vs. 1-2)	0.49 (0.16,1.55)	0.226	-	-
Combination therapy (Yes vs. No)	0.46 (0.16,1.33)	0.154	0.5 (0.17,1.47)	0.209

**Table 4:** Univariate and multivariate analyses of factors for overall survival in the combined two cohorts.

Variables	Univariate analysis		Multivariate analysis	
	HR (95% CI)	*p*	HR (95% CI)	*p*
Sex (Female vs. Male)	0.79 (0.15,4.15)	0.783	-	-
Age (≥60 vs. <60)	0.98 (0.19,5.1)	0.985	-	-
Smoking history (Yes vs. No)	28.31 (2.35,340.82)	0.008	30.74 (2.11,447.25)	0.012
TNM stage (IV vs. III)	2.41 (0.28,20.51)	0.42	-	-
PD-L1 expression (Low vs. High)	0.59 (0.11,3.11)	0.538	-	-
Multiple metastases (Yes vs. No)	3.26 (0.79,13.51)	0.103	1.83 (0.38,8.69)	0.449
Liver metastasis (Yes vs. No)	3.07 (0.68,13.77)	0.144	3.6 (0.68,19.21)	0.133
Treatment line (>2 vs. 1-2)	0.58 (0.13,2.54)	0.467	-	-
Combination therapy (Yes vs. No)	0.22 (0.01,14.6)	0.249	-	-

## Discussions

Pulmonary LELC is a rare subtype of NSCLC characterized by EBV infection and abundant lymphocyte infiltration. EBV infection has been linked to upregulation of PD-L1 expression in malignancies (24). On the other hand, a high level of lymphocyte infiltration and PD-L1 expression are believed to be associated with immunotherapy response (25). However, the efficacy of PD-1/PD-L1 inhibitors in pulmonary LELC needs to be further verified.

In the present study, we described 10 previously treated LELC patients receiving PD-1 inhibitors, with an ORR as high as 62.5%. Of note, in a retrospective study previously reported by our institution, 41 LELC patients treated by 2^nd^ or above chemotherapy had an ORR of 20-25% (6). Likewise, previously treated LELC patients receiving immunotherapy also tended to have longer PFS and OS (6). Furthermore, we performed a literature review, and found that pulmonary LELC tends to have a higher PD-L1 expression and desirable immunotherapy response compared with other subtypes of NSCLC (26). Consistently, in the CHCSJ cohort, our results suggested that the PFS of patients with PD-L1 TPS >30% was significantly longer than those with TPS ≤30%. In contrast, the association of prolonged PFS with higher PD-L1 expression was not observed in patients retrieved from literature or when two cohorts were combined. Furthermore, the prognostic significance (both PFS and OS) of PD-L1 expression levels in LELC patients treated with chemotherapy is also controversial (27–29). Intriguingly, when we focused on the patients of Chinese descent, those with high PD-L1 expression (TPS >30%) tended to have a prolonged PFS (n=29, p=0.09, data not shown). Even when the study was limited to the patients who received PD-1/PD-L1 inhibitors in China, high PD-L1 expression was significantly associated with prolonged PFS (n=28, p=0.049, data not shown). These results suggest the possibility that patient pedigree and environmental factors may influence the efficacy of immunotherapy in pulmonary LELC. On all accounts, given the desirable efficacy of PD-1/PL-L1 inhibitors in lung and nasopharyngeal cancer, our results support the administration of immunotherapy in patients with pulmonary LELC.

Notably, our results suggested non-smoking patients with pulmonary LELC may have better survival outcomes when compared to ever-smokers. However, the effect of smoking on the tumor immune microenvironment is complicated. On the one hand, smoking has been associated with elevated tumor mutation burden and PD-L1 expression, suggesting better immunotherapy results (30). On the other hand, smoking may impair PD-1/PD-L1 response by inhibiting immune cell infiltration into tumors (31). Studies support that smoking can improve the efficacy of checkpoint inhibitor monotherapy, but does not significantly affect the response of NSCLC to immunochemotherapy combination (32). Specifically, a previous study reported that smoking was an independent predictor of unfavorable survival in patients with pulmonary LELC (33). Overall, the effect of smoking status on immunotherapy outcomes in patients with LELC needs to be further confirmed in larger cohorts.

To the best of our knowledge, the present study reports the largest cohort to date of pulmonary LELC patients treated with PD-1 inhibitors, and our results support the administration of immunotherapy in LELC patients. Nevertheless, several limitations need to be highlighted. Firstly, the relatively small scale of the CHCSJ and literature review cohorts might lead to selection bias. Although we did a thorough screening in Pubmed, only 1 case whose disease progressed after the application of nivolumab was found. This may be the case, meaning the vast majority of advanced pulmonary LELCs responded well or at least with stable disease to anti-PD-1/PD-L1 therapy, or cases that didn’t benefit from immunotherapy hadn’t been reported and included in this study. Unavailability of the responses in several cases was another reason why the efficacy of anti-PD-1/PD-L1 antibodies should not be overestimated in daily practice. Secondly, some of the public data were lacking. For example, patient fitness and treatment-related adverse events were untouched in this study due to incomplete data and it may have an impact on clinical outcomes of pulmonary LELCs. PD-L1 expressions in some cases were inaccessible and different antibodies such as SP142 or 22C3 were used for TPS evaluation in public data. All may interfere with our endeavor to identify the relationship between PD-L1 expression and the efficacy of anti-PD-1/PD-L1 antibodies, for blueprint study showed interchangeability of 22C3 and other antibodies but lower sensitivity of SP142 in NSCLC TPS IHC assay (34). Thirdly, the study was puzzled by various anti-PD-1/PD-L1 regimens and their combinations with chemotherapy or targeted therapy. However, all these PD-1/PD-L1 inhibitors have been proven to be comparably effective in treating driver mutation-negative NSCLC either on their own or joined by other active agents (35). Nevertheless, combining public data and Macau cases, our analysis made a good summary of how PD-1/PD-L1 antagonists performed in LELCs and proved a positive correlation between PD-L1 expression on the efficacy of immunotherapy. It also raised the evidence of using PD-1/PD-L1 inhibitors in advanced pulmonary LELC to a higher level rather than individual experiences.

In conclusion, our results preliminarily examine the efficacy of anti-PD-1 antibodies in patients with pulmonary LELC. Further validation was also warranted for solid conclusions supplementary to the scarce specified data about immunotherapy in pulmonary LELC.

## Data availability statement

The raw data supporting the conclusions of this article will be made available by the authors, without undue reservation.

## Ethics statement

The studies involving human participants were reviewed and approved by the Ethics Committee of CHCSJ. Written informed consent for participation was not required for this study in accordance with the national legislation and the institutional requirements.

## Author contributions

NZ, YYW and YZW conceived the study. NZ, HT, YL and YZW reviewed medical records and analyzed the data. SY performed pathological review. NZ, HT and YZW drafted the manuscript. YYW revised the draft. All authors contributed to the article and approved the submitted version.
