# Supplementary material for: Anti-PD-1 antibodies, a novel treatment option for advanced chemoresistant pulmonary lymphoepithelioma carcinoma
Source: Front Immunol. 2022 Dec 6;13:1001414. doi: 10.3389/fimmu.2022.1001414 (PMC9763302; doi:10.3389/fimmu.2022.1001414)
Supplement: Supplementary file 1 [file DataSheet_1.docx]

**Supplementary materials**


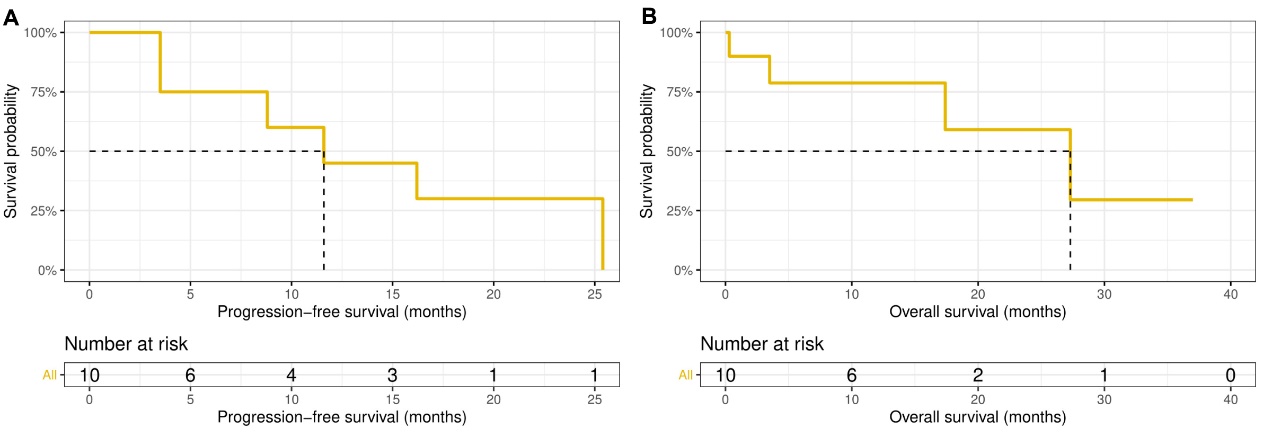


Figure S1. Kaplan-Meier curves of progression-free survival (A) and overall survival (B) in the CHCSJ cohort.


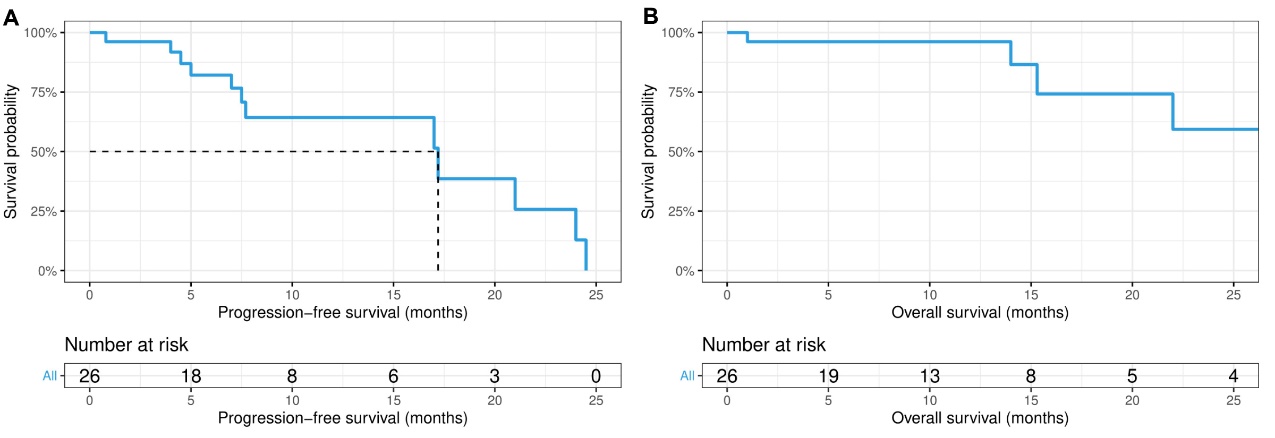


Figure S2. Kaplan-Meier curves of progression-free survival (A) and overall survival (B) in the patients retrieved from the literature review.


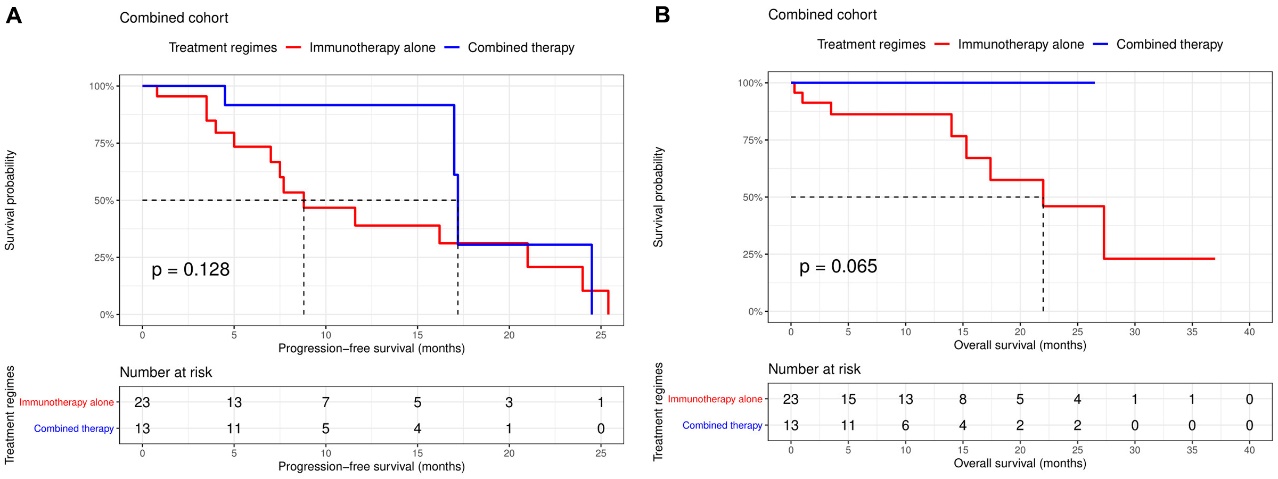
 Figure S3. Comparison of progression-free survival (A) and overall survival (B) between patients receiving immunotherapy alone and immunotherapy combined with chemotherapy or targeted therapy in the combined cohorts.
